# Humans as Reservoir for Enterotoxin Gene–carrying *Clostridium perfringens* Type A

**DOI:** 10.3201/eid1211.060478

**Published:** 2006-11

**Authors:** Annamari Heikinheimo, Miia Lindström, Per Einar Granum, Hannu Korkeala

**Affiliations:** * University of Helsinki, Helsinki, Finland;; † Norwegian School of Veterinary Science, Oslo, Norway

**Keywords:** *cpe*, *Clostridium perfringens*, epidemiology, feces, type A, plasmid, chromosome, research

## Abstract

We found a prevalence of 18% for enterotoxin gene–carrying (*cpe*+) *Clostridium perfringens* in the feces of healthy food handlers by PCR and isolated the organism from 11 of 23 PCR-positive persons by using hydrophobic grid membrane filter-colony hybridization. Several different *cpe* genotypes were recovered. The prevalence was 3.7% for plasmidial IS*1151*-*cpe*, 2.9% for plasmidial IS*1470*-like-*cpe*, 0.7% for chromosomal IS*1470-cpe*, and 1.5% for unknown *cpe* genotype. Lateral spread of *cpe* between *C. perfringens* strains was evident because strains from the same person carried IS*1470*-like *cpe* but shared no genetic relatedness according to pulsed-field gel electrophoresis analysis. Our findings suggest that healthy humans serve as a rich reservoir for *cpe*+ *C. perfringens* type A and may play a role in the etiology of gastrointestinal diseases caused by this organism. The results also indicate that humans should be considered a risk factor for spread of *C. perfringens* type A food poisoning and that they are a possible source of contamination for *C. perfringens* type A food poisoning.

## Abstract 2

Humans may play a role in the transmission of gastrointestinal diseases caused by *C. perfringens*.

Clostridium perfringens is classified into 5 types (A–E) on the basis of its ability to produce >1 of the major lethal toxins α, β, ε, and ι (*1*). Enterotoxin (CPE)-producing (cpe+) C. perfringens type A is reported continuously as 1 of the most common food poisoning agents worldwide (*2**–**4*). An increasing number of reports also implicate the organism in 5%–15% of antimicrobial drug–associated diarrhea (AAD) and sporadic diarrhea (SD) cases in humans as well as diarrhea cases in animals (*1**,**5**–**9*). Most food poisoning strains studied to date carry cpe in their chromosomes; isolates from AAD and SD cases bear cpe in a plasmid (*10**,**11*). Furthermore, genetic studies have shown that in C. perfringens strains with the chromosomally located cpe, IS1470 sequences are found upstream and downstream of cpe (*12**,**13*). However, in strains with cpe in the plasmid, 2 different genetic arrangements (either IS1151 or IS1470-like sequences) have been recognized downstream of cpe (*11**,**14*).

Why C. perfringens strains with cpe located on chromosomes or plasmids cause different diseases has not been satisfactorily explained. However, the relatively greater heat resistance of the strains with chromosomally located cpe is a plausible explanation for these strains' survival in cooked food, thus causing instances of food poisonings (*15*). The presence of C. perfringens strains with chromosomally located cpe in 1.4% of American retail food indicates that these strains have an access to the food chain (*16*). The sources and routes of contamination are unclear.

An explanation for the strong association between C. perfringens strains with plasmidially located cpe and cases of AAD and SD disease may be in vivo transfer of the cpe plasmid to C. perfringens strains of the normal intestinal microbiota (*17*). Thus, a small amount of ingested cpe+ C. perfringens would act as an infectious agent and transfer the cpe plasmid to cpe– C. perfringens strains of the normal microbiota. This process would result in the persistence of cpe+ C. perfringens in the intestines. Chronic exposure to CPE would explain the severity and long duration of symptoms (*17*). Conjugative transfer of the cpe plasmid has been demonstrated in vitro (*18*), but currently no data exist on lateral transfer of cpe in vivo, and whether cpe+ strains that cause AAD and SD are resident in the gastrointestinal tract or acquired before onset of the disease is unknown.

Although the ubiquitous distribution of C. perfringens in nature is well documented, the epidemiology of cpe+ strains has not yet been established. Less than 5% of global C. perfringens isolates are estimated to carry cpe (*1*), and the prevalence of different cpe genotypes in specific ecologic niches is not known. In this study, healthy persons were screened for fecal carriage of cpe, and cpe+ strains were further isolated by using hydrophobic grid membrane filter-colony hybridization. The cpe genotype and location of cpe were determined from strains by detecting different insertion sequence (IS) elements attached to cpe (plasmid types IS1151-cpe and IS1470-like-cpe and chromosomal type IS1470-cpe). The genetic relationship between cpe+ and cpe– C. perfringens isolates obtained from cpe carriers was assessed with pulsed-field gel electrophoresis (PFGE).

## Materials and Methods

### Fecal Samples

A total of 136 fecal samples, 102 (75%) from female food handlers and 34 (25%) from male food handlers, were collected from food handlers in southern Finland during summer 2003. These persons reported no gastrointestinal symptoms at the time of sampling. Their ages ranged from 15 to 65 years (mean 30 years, median 24 years). The samples were kept at –70°C until investigated.

### Detection and Isolation

Each fecal sample (1 g) was divided into 2 tubes that contained freshly prepared thioglycollate (40 mL) (Oxoid, Basingstoke, UK). One tube was heated at 75°C for 20 min; the other was left unheated. After anaerobic incubation at 37°C for 20–22 h, the presence of cpe in each tube was determined by nested PCR (*19*). Persons with a cpe-positive fecal sample are hereafter referred to as cpe carriers.

Hydrophobic grid membrane filter-colony hybridization (HGMF-CH) (*20*) was used to isolate cpe+ C. perfringens from cpe carriers. In addition to probe-positive colonies, those showing no hybridization signals but having a typical color for C. perfringens colonies (usually black, occasionally gray or grayish yellow) were isolated from each sample to obtain a collection of both cpe+ and cpe– C. perfringens from a single sample and to further study the genetic relatedness of these isolates by PFGE (see below).

The isolates were subjected to PCR to determine the toxinotype (A–E) and the presence of cpe (*21*). C. perfringens strains NCTC 8239, ATCC 3626, CCUG 2036, CCUG 2037, and CCUG 44727 were used as positive controls.

### Molecular Typing

C. perfringens isolates that possessed cpe were further studied by PCR to determine the cpe genotype of the strain. Total DNA was isolated by using Advamax beads (Edge Biosystems, Gaithersburg, MD, USA) according to the manufacturer's instructions. IS elements downstream of cpe that determine the cpe genotype (IS1151-cpe, IS1470-like-cpe, or IS1470-cpe) of each isolate were characterized by using primers and protocols described in Table 1. The location of cpe (plasmid or chromosome) was concluded according to the genotyping results (Table 1).

**Table 1:** Primers and PCR protocols for determining the genotype and location of cpe*






Primer	Sequence (5´ to 3´)	Primer target	Sequence ref, GenBank accession no.	Ref	Size	*cpe* genotype	*cpe* location

CPEmmF	CAAGTCAAATTCTTAATCCT	*cpe*	(*22*), Y16009	(*12*)	1.2 kb	IS1151-cpe	Plasmid
*1151* R	CATGGCCGTCAACCTAAGAAG	IS*1151*	(*23*), X60694				
CPEmmF	CAAGTCAAATTCTTAATCCT	*cpe*	(*22*), Y16009	(*12*)	2.4 kb	IS1470-cpe	Chromosome
*1470*mR	TGAAAACCGTGAAGAATTTGG	IS*1470*	(*12*), X71844				
cpe4F	TTAGAACAGTCCTTAGGTGATGGAG	*cpe*	(*14*), AF511071	(*24*)	1.6 kb	IS1470-like-cpe	Plasmid
IS*1470*-likeR1.6	CTTTGTGTACACAGCTTCGCCAATGTC	IS*1470*-like	(*24*), AF416450				


*Ref, reference.

C. perfringens isolates were then typed by PFGE with ApaI and SmaI (New England Biolabs, Beverly, MA, USA) to study the genetic relationships between isolates (*25*). PFGE profiles were analyzed visually and with a computer software program (Bionumerics, version 4.5; Applied Maths, Kortrijk, Belgium). The similarities between macrorestriction patterns (MRP) were expressed by Dice coefficient correlation, and clustering by the unweighted pair-group method with arithmetic averages was used to construct a dendrogram.

### Cytotoxicity Test on Vero Cells

To test the capability of cpe+ strains to produce CPE, 1–3 cpe+ isolates representing each MRP were sporulated (*26*). The final culture in modified Duncan-Strong medium (Sigma-Aldrich Chemie, Steinheim, Switzerland) was examined by phase-contrast microscopy to confirm the sporulation of the strain. The culture was then sonicated until >95% of the spores were free, as determined by phase-contrast microscopy. The culture was centrifuged at 1,500× g for 25 min at 4°C, and cytotoxicity of the supernatant was tested with a Vero cell assay according to Sandvig and Olsnes (*27*). The assay monitors the inhibition of protein synthesis in Vero cells after addition of toxic proteins, including CPE. The Vero cells were grown in a minimal essential medium (Gibco BRL, Paisley, UK) supplemented with 10% fetal calf serum.

The supernatant was precipitated in 80% saturated ammonium sulfate and kept at 4°C before the Vero cell assay. The strain was defined as producing CPE when the inhibition of protein synthesis of Vero cells was >20%.

## Results

### Detection and Isolation

The gene encoding for cpe was detected in the feces of 25 food handlers (18%). For 23 samples (92%), the heated tube showed the positive result, and for 3 of them the unheated tube also yielded a positive PCR result. For 2 samples, only the unheated tube showed a positive PCR result. No association between gender or age and carrier status of cpe was found (Table 2).

**Table 2:** Association of sex and age with *cpe* in feces of healthy humans




Characteristic	*cpe* detected by PCR/total no. fecal samples (%)	

Sex		
Female	19/102 (18.6)	
Male	6/34 (17.6)	
Total	25/136 (18.4)	
Age, y		
<20	6/40 (15.0)	
20–50	16/79 (20.3)	
>50	3/17 (17.6)	
Total	25/136 (18.4)	



The HGMF-CH method was used to determine whether cpe+ C. perfringens was present in 23 persons; isolation was successful from 11 persons. The average number of cpe+ C. perfringens isolates carried by these persons was 2.5 × 10^2^ CFU/g. In 10 of these persons, cpe– C. perfringens was also recovered; the average number was 1.8 × 10^4^ CFU/g. In each of these 10 case-patients, cpe– C. perfringens formed a 10- to 1,000-fold majority of the C. perfringens population. In 1 person, only cpe+ C. perfringens isolates were recovered. A total of 77 C. perfringens isolates (average of 7 isolates per person) were recovered; all possessed the gene encoding for the α toxin only, which signified that they belonged to type A (Table A1). Of these, 36 isolates were positive for cpe (Table A1).

### Molecular Typing

**Figure 1:**
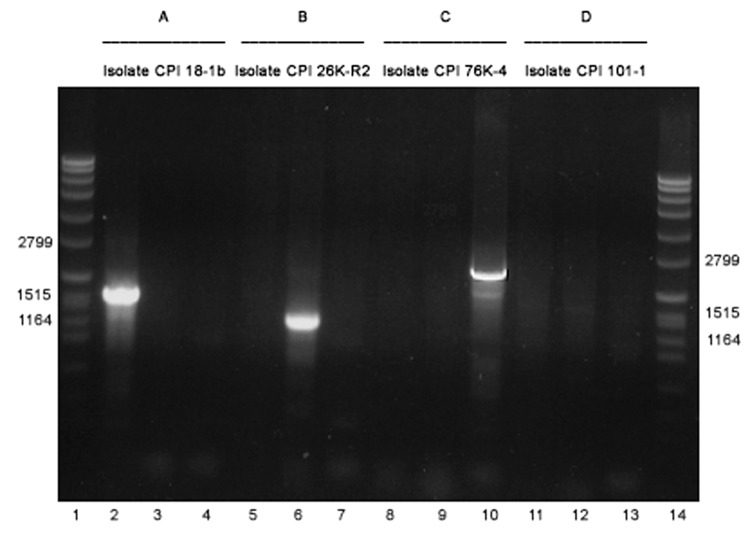
PCR analysis determining the genotype of enterotoxin gene–carrying *Clostridium perfringens* type A isolates obtained from healthy persons. All 4 strains are studied with 3 different primer sets, described in Table 1. For A, B, C and D, the isolate represents genotype *IS1470-like-cpe*, *IS1151-cpe*, *IS1470-cpe*, or unknown genotype, respectively.

When the relative prevalence of different cpe genotypes was determined with PCR, strains representing the IS1151-cpe type were found in 5 persons (3.7%) and strains representing the IS1470-like-cpe type were found in 4 persons (2.9%), findings that indicated that all of these persons carried C. perfringens strains with plasmidially located cpe. A strain with chromosomally located cpe, representing the IS1470-cpe type, was detected in 1 person (0.7%). Furthermore, cpe+ C. perfringens strains representing none of the aforementioned types, referred to as cpe+ strains with an unknown genetic arrangement downstream of cpe, were observed in 2 persons (1.5%) (Table A1) (Figure 1).

**Figure 2:**
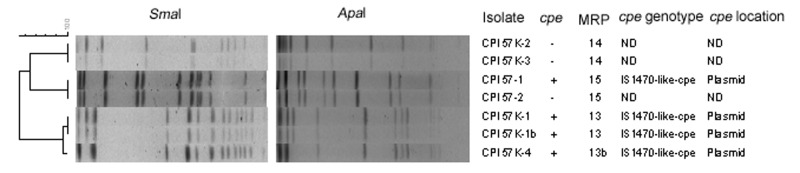
Pulsed-field gel electrophoresis analysis and determination of the *cpe* genotype of *Clostridium perfringens* isolates obtained from a healthy person. ND, not determined.

In PFGE analysis, all isolates were typeable with SmaI restriction enzyme, and all but 2 isolates were typeable with ApaI. The discriminatory power was equal with both enzymes used, which showed a high genetic diversity among C. perfringens. PFGE analysis showed 1–5 different MRPs in each person, with cpe+ and cpe– isolates generally being unrelated to each other. However, in 1 case, 2 isolates (CPI 57–1 and CPI 57–2) from the same person showed identical MRPs with both restriction enzymes, the former being cpe+ and the latter cpe– (Table A1; Figure 2). In 8 of 11 persons, cpe+ isolates with similar MRPs were identified, whereas 3 persons carried cpe+ isolates with 2 different MRPs. In 2 of these persons (numbers 18 and 57), both MRPs represented the same cpe genotype (IS1470-like-cpe); the third person (number 75) had MRPs with different cpe genotypes (IS1470-like-cpe and IS1151-cpe) (Table A1).

### Cytotoxicity Test on Vero Cells

Successful sporulation was achieved with isolates representing 9 different MRPs. In 8 (89%) of these MRPs, >20% inhibition of protein synthesis was detected in Vero cells, which suggested CPE production of the strain (Table 3).

**Table 3:** Cytotoxicity test on Vero cells to determine the production of CPE by *cpe*+ *C. perfringens* strains obtained from healthy persons

Isolate	MRP*	CPE†	*cpe* genotype	*cpe* location

CPI 18–3	1	+	IS*1470*-like-*cpe*	Plasmid
CPI 18–2	2	NS	IS*1470*-like-*cpe*	Plasmid
CPI 26K-R3p	5	+	IS*1151*-*cpe*	Plasmid
CPI 39–1b, CPI 39K-7	6	+	IS*1470*-like-*cpe*	Plasmid
CPI 44K-R3, CPI 44K-R7	10	NS	Untypeable	Not known
CPI 53K-R3	11	NS	IS*1151*-*cpe*	Plasmid
CPI 57K-1	13	+	IS*1470*-like-*cpe*	Plasmid
CPI 57–1	15	NS	IS*1470*-like-*cpe*	Plasmid
CPI 63K-R5	19	NS	IS*1151*-*cpe*	Plasmid
CPI 75–3a, CPI 75–3b, CPI 75–5b	21	-	IS*1151*-*cpe*	Plasmid
CPI 75–4	22	+	IS*1470*-like-*cpe*	Plasmid
CPI 76K-4, CPI 76K-5	24	+	IS*1470-cpe*	Chromosome
CPI 101–4	25	+	Untypeable	Not known
CPI 103K-3, CPI 103K-5, CPI 103K-6	29	+	IS*1151*-*cpe*	Plasmid

*MRP, macro restriction pattern. †+, present; –, absent, NS, not sporulated and thus cytotoxicity test on Vero cells not performed.				

## Discussion

We report the first in-depth study of the fecal carriage of cpe+ C. perfringens by healthy humans, which showed that the organism is widely distributed in this ecologic niche. By using the novel HGMF-CH method, we demonstrated that low numbers of cpe+ C. perfringens strains are frequently present among the dominant cpe– C. perfringens. HGMF-CH proved invaluable in the isolation of cpe+ C. perfringens; in all instances, when both cpe+ and cpe– C. perfringens were isolated, the former existed as a minority and thus would have been missed by conventional isolation methods.

Healthy persons are a rich reservoir for cpe+ C. perfringens; type A strains representing several different cpe genotypes (strains with plasmidially located IS1470-like–cpe or IS1151-cpe and chromosomally located IS1470-cpe) as well as type A strains with unrecognized genetic arrangement attached to cpe were present. That IS1151-cpe was the most prevalent and IS1470-cpe represented the minority of the cpe genotypes are findings in line with a previous study that suggested that cpe+ C. perfringens strains with plasmidially located cpe are more common in nature than cpe+ C. perfringens strains with chromosomally located cpe (*28*). The presence of strains with unrecognized genetic arrangement attached to cpe reflects the wide genetic variety of cpe+ strains. Because production of CPE was demonstrated in 1 of these cpe+ strains with an unknown cpe genotype, the gene was apparently intact and functional.

The presence of cpe+ C. perfringens type A strain with chromosomally located cpe and a full capacity to produce CPE in the feces of healthy food handlers indicates that human handling of food should be considered a risk factor for contamination. C. perfringens type A food poisoning typically follows from the ingestion of cpe+ C. perfringens vegetative cells formed in food during storage and serving (*29*). The low numbers of cpe+ C. perfringens spores present in the feces of the person handling the food may be transferred to the food. Under favorable conditions, cpe+ strains with chromosomally located cpe will easily survive and multiply during food processing and cause food poisoning because they survive broader temperature ranges during growth and maintenance phases than other C. perfringens strains (*15**,**30*).

Evidence of in vivo horizontal transfer of cpe between C. perfringens strains was obtained in the PFGE analysis. First, in 2 persons (numbers 18 and 57), IS1470-like-cpe was observed in strains with no genetic relatedness, which indicated lateral spread of IS1470-like-cpe (Table A1). In vitro conjugative transfer of the cpe plasmid has been demonstrated with strain F4969 carrying IS1470-like-cpe (*18**,**24*), which supports our findings. Second, the evidence of in vivo horizontal transfer of cpe was further strengthened by observing a loss or acquisition of IS1470-like-cpe i n 1 strain (MRP 15) (Table A1). In this case, the isolate CPI 57–1 carried IS1470-like-cpe, whereas CPI 57–2 was lacking in the same element; these strains were nevertheless isolated from the same person and shared an identical MRP. A potential donor or recipient strain (MRP 13 and 13b) was demonstrated from the same sample, which carried IS1470-like-cpe but shared no genetic relatedness to CPI 57–1 (Table A1) (Figure 2). All IS1151-cpe isolates from the same persons shared identical MRPs; thus, no evidence for in vivo lateral transfer of IS1151-cpe was observed.

Finally, our study provides a new insight into the pathogenesis of AAD and SD caused by cpe+ C. perfringens type A. These diseases have been speculated to result from the ingestion of small numbers of cpe+ strains, which transfer the cpe plasmid to cpe– C. perfringens strains present in the normal intestinal microbiota (*17*). Our results support this theory because we found small numbers of cpe+ C. perfringens type A with plasmidially located cpe in the human gastrointestinal tract as well as evidence for the lateral spread of cpe. Our findings therefore indicate that AAD or SD caused by cpe+ C. perfringens type A may occur as an endogenous infection, present in the gastrointestinal tract and causing the disease after exposure to antimicrobial drugs or other predisposing factors. However, the capacity of these cpe+ strains to persist in the gastrointestinal tract remains unknown; as does how these strains find their way to the gastrointestinal tract. Nevertheless, because they are apparently common in human feces, cpe+ strains are presumably acquired from the environment. The question then arises whether these cpe+ strains are ingested with food, which would indicate that AAD and SD caused by cpe+ C. perfringens type A are foodborne.

In conclusion, healthy humans serve as a rich reservoir for cpe+ C. perfringens type A strains and may play an important role in gastrointestinal diseases caused by this pathogen. Humans should therefore be considered a risk factor for spread of C. perfringens type A food poisoning. Future studies to determine the presence of different cpe genotypes in other ecologic niches are warranted to elucidate the epidemiology of this major pathogen.

## Appendix

**Table A1:** Characterization of *Clostridium perfringens* isolates from fecal samples of healthy humans, type A*

Subject	Isolate	*cpe*	MRP	Genotyping PCR	*cpe* genotype	*cpe* location

CPEmmF and 1151R	CPEmmF and 1470mR	cpe4F and IS1470-like R1.6

18	CPI 18–1b	+	1	–	–	+	IS*1470*-like-*cpe*	Plasmid
	CPI 18–2	+	2	–	–	+	IS*1470*-like-*cpe*	Plasmid
	CPI 18–3	+	1	–	–	+	IS*1470*-like-*cpe*	Plasmid
	CPI 18–4	+	2b	–	–	+	IS*1470*-like-*cpe*	Plasmid
	CPI 18–5	+	2b	–	–	+	IS*1470*-like-*cpe*	Plasmid
	CPI 18–6	–	3	ND	ND	ND	ND	ND

26	CPI 26K-R1	–	4	ND	ND	ND	ND	ND
	CPI 26K-R2	+	5	+	–	–	IS*1151*-*cpe*	Plasmid
	CPI 26K-R3	+	5	+	–	–	IS*1151*-*cpe*	Plasmid
	CPI 26K-R3p	+	5	+	–	–	IS*1151*-*cpe*	Plasmid

39	CPI 39–1a	+	6	–	–	+	IS*1470*-like-*cpe*	Plasmid
	CPI 39–1b	+	6	–	–	+	IS*1470*-like-*cpe*	Plasmid
	CPI 39–2a	–	7	ND	ND	ND	ND	ND
	CPI 39–2b	–	7	ND	ND	ND	ND	ND
	CPI 39K-1a	–	8	ND	ND	ND	ND	ND
	CPI 39K-1b	–	7	ND	ND	ND	ND	ND
	CPI 39K-2a	–	7	ND	ND	ND	ND	ND
	CPI 39K-2b	–	7	ND	ND	ND	ND	ND
	CPI 39K-4a	–	7	ND	ND	ND	ND	ND
	CPI 39K-4b	–	7	ND	ND	ND	ND	ND
	CPI 39K-7	+	6	–	–	+	IS*1470*-like-*cpe*	Plasmid
	CPI 39K-8	–	7	ND	ND	ND	ND	ND

44	CPI 44K-R1	–	9	ND	ND	ND	ND	ND
	CPI 44K-R2	–	9	ND	ND	ND	ND	ND
	CPI 44K-R3	+	10	–	–	–	Untypeable	Not known
	CPI 44K-R4	–	9	ND	ND	ND	ND	ND
	CPI 44K-R5	–	9	ND	ND	ND	ND	ND
	CPI 44K-R6	–	9	ND	ND	ND	ND	ND
	CPI 44K-R7	+	10	–	–	–	Untypeable	Not known
	CPI 44K-R8	–	9	ND	ND	ND	ND	ND

53	CPI 53K-R1	+	11	+	–	–	IS*1151*-*cpe*	Plasmid
	CPI 53K-R2	–	12	ND	ND	ND	ND	ND
	CPI 53K-R3	+	11	+	–	–	IS*1151*-*cpe*	Plasmid
	CPI 53K-R4	–	12	ND	ND	ND	ND	ND
	CPI 53K-R5	–	12	ND	ND	ND	ND	ND
	CPI 53K-R6	–	12	ND	ND	ND	ND	ND
	CPI 53K-R7	+	11	+	–	–	IS*1151*-*cpe*	Plasmid

57	CPI 57K-1	+	13	–	–	+	IS*1470*-like-*cpe*	Plasmid
	CPI 57K-1b	+	13	–	–	+	IS*1470*-like-*cpe*	Plasmid
	CPI 57K-2	–	14	ND	ND	ND	ND	ND
	CPI 57K-3	–	14	ND	ND	ND	ND	ND
	CPI 57K-4	+	13b	–	–	+	IS*1470*-like-*cpe*	Plasmid
	CPI 57–1	+	15	–	–	+	IS*1470*-like-*cpe*	Plasmid
	CPI 57–2	–	15	–	–	–	ND	ND

63	CPI 63K-R1	–	16	ND	ND	ND	ND	ND
	CPI 63K-R2	–	17	ND	ND	ND	ND	ND
	CPI 63K-R3	–	17	ND	ND	ND	ND	ND
	CPI 63K-R4	–	18	ND	ND	ND	ND	ND
	CPI 63K-R5	+	19	+	–	–	IS*1151*-*cpe*	Plasmid
	CPI 63K-R6	–	20	ND	ND	ND	ND	ND

75	CPI 75–1	+	21	+	–	–	IS*1151*-*cpe*	Plasmid
	CPI 75–3a	+	21	+	–	–	IS*1151*-*cpe*	Plasmid
	CPI 75–3b	+	21	+	–	–	IS*1151*-*cpe*	Plasmid
	CPI 75–4	+	22	–	–	+	IS*1470*-like-*cpe*	Plasmid
	CPI 75–6	–	23	ND	ND	ND	ND	ND
	CPI 75K-2a	+	21	+	–	–	IS*1151*-*cpe*	Plasmid
	CPI 75K-2b	+	21	+	–	–	IS*1151*-*cpe*	Plasmid
	CPI 75K-3	+	21b	+	–	–	IS*1151*-*cpe*	Plasmid
	CPI 75K-4	+	22b	–	–	+	IS*1470*-like-*cpe*	Plasmid
	CPI 75K-7	–	23	ND	ND	ND	ND	ND
	CPI 75K-8	–	23	ND	ND	ND	ND	ND

76	CPI 76K-4	+	24	–	+	–	IS*1470*-*cpe*	Chromosome
	CPI 76K-5	+	24	–	+	–	IS*1470*-*cpe*	Chromosome

101	CPI 101–1	+	25	–	–	–	Untypeable	Not known
	CPI 101–2	–	26	ND	ND	ND	ND	ND
	CPI 101–3a	–	27	ND	ND	ND	ND	ND
	CPI 101–3b	–	27	ND	ND	ND	ND	ND
	CPI 101–4	+	25	–	–	–	Untypeable	Not known
	CPI 101–5	–	26	ND	ND	ND	ND	ND

103	CPI 103K-1	–	28	ND	ND	ND	ND	ND
	CPI 103K-2	–	28	ND	ND	ND	ND	ND
	CPI 103K-3	+	29	+	–	–	IS*1151*-*cpe*	Plasmid
	CPI 103K-4	–	28	ND	ND	ND	ND	ND
	CPI 103K-5	+	29	+	–	–	IS*1151*-*cpe*	Plasmid
	CPI 103K-6	+	29	+	–	–	IS*1151*-*cpe*	Plasmid
	CPI 103K-7	–	28	ND	ND	ND	ND	ND
	CPI 103K-8	–	28	ND	ND	ND	ND	ND

*MRP, mutlidrug resistance–associated protein; Not known means that the *cpe* gene is present in the isolate but the location (plasmid/chromosome) of the gene is not known. ND means that since the *cpe* gene is not present in the isolate, the location of the gene cannot naturally be determined.
